# Supplementary material for: Bacillus velezensis FX-6 suppresses the infection of Botrytis cinerea and increases the biomass of tomato plants
Source: PLoS One. 2023 Jun 15;18(6):e0286971. doi: 10.1371/journal.pone.0286971 (PMC10270589; doi:10.1371/journal.pone.0286971)
Supplement: S1 File — (PDF) [file pone.0286971.s001.pdf]

>FX-6(16srDNA)

CTTCTCCGTCGTGAGACTACTATACTATACATGCAAGTCGAGCGGACAGATGGGAGCTTGCTCCCTGATGTTA
GCGGCGGACGGGTGAGTAACACGTGGGTAACCTGCCTGTAAGACTGGGATAACTCCGGGAAACCGGGGCT
AATACCGGATGGTTGTTTGAACCGCATGGTTCAGACATAAAAGGTGGCTTCGGCTACCACTTACAGATGGAC
CCGCGGCGCATTAGCTAGTTGGTGAGGTAACGGCTCACCAAGGCAACGATGCGTAGCCGACCTGAGAGGG
TGATCGGCCACACTGGGACTGAGACACGGCCCAGACTCCTACGGGAGGCAGCAGTAGGGAATCTTCCGCA
ATGGACGAAAGTCTGACGGAGCAACGCCGCGTGAGTGATGAAGGTTTTCGGATCGTAAAGCTCTGTTGTTA
GGGAAGAACAAGTGCCGTTCAAATAGGGCGGCACCTTGACGGTACCTAACCAGAAAGCCACGGCTAACTA
CGTGCCAGCAGCCGCGGTAATACGTAGGTGGCAAGCGTTGTCCGGAATTATTGGGCGTAAAGGGCTCGCAG
GCGGTTTCTTAAGTCTGATGTGAAAGCCCCCGGCTCAACCGGGGAGGGTCATTGGAAACTGGGGAACTTG
AGTGCAGAAGAGGAGAGTGGAATTCCACGTGTAGCGGTGAAATGCGTAGAGATGTGGAGGAACACCAGT
GGCGAAGGCGACTCTCTGGTCTGTAACTGACGCTGAGGAGCGAAAGCGTGGGGAGCGAACAGGATTAGAT
ACCCTGGTAGTCCACGCCGTAAACGATGAGTGCTAAGTGTTAGGGGGTTTCCGCCCCTTAGTGCTGCAGCTA
ACGCATTAAGCACTCCGCCTGGGGAGTACGGTCGCAAGACTGAAACTCAAAGGAATTGACGGGGGCCCGC
ACAAGCGGTGGAGCATGTGGTTTAATTCGAAGCAACGCGAAGAACCTTACCAGGTCTTGACATCCTCTGAC
AATCCTAGAGATAGGACGTCCCCTTCGGGGGCAGAGTGACAGGTGGTGCATGGTTGTCGTCAGCTCGTGTC
GTGAGATGTTGGGTTAAGTCCCGCAACGAGCGCAACCCTTGATCTTAGTTGCCAGCATTCAGTTGGGCACTC
TAAGGTGACTGCCGGTGACAAACCGGAGGAAGGTGGGGATGACGTCAAATCATCATGCCCCTTATGACCTG
GGCTACACACGTGCTACAATGGACAGAACAAAGGGCAGCGAAACCGCGAGGTTAAGCCAATCCCACAAAT
CTGTTCTCAGTTCGGATCGCAGTCTGCAACTCGACTGCGTGAAGCTGGAATCGCTAGTAATCGCGGATCAGC
ATGCCGCGGTGAATACGTTCCCGGGCCTTGTACACACCGCCCGTCACACCACGAGAGTTTGTAACACCCGAA
GTCGGTGAGGTAACCTTTAGGAGCCAGCCGCCGAATTGTGAACTTCACCCCTTAT
