# Supplementary material for: Bacillus velezensis FX-6 suppresses the infection of Botrytis cinerea and increases the biomass of tomato plants
Source: PLoS One. 2023 Jun 15;18(6):e0286971. doi: 10.1371/journal.pone.0286971 (PMC10270589; doi:10.1371/journal.pone.0286971)
Supplement: S2 File — (PDF) [file pone.0286971.s002.pdf]

>FX-6(gyrA)
AGTCAGGAAAATGCGTACGTCCTTTCTGGACTATGCAATGAGCGTTATCGTATCCCGGGCGCTTCCGGATGT
GCGTGACGGTCTGAAGCCGGTTCACAGGCGGATTTTGTACGCAATGAATGATTTAGGCATGACCAGTGACA
AACCATATAAAAAATCTGCCCGTATCGTCGGTGAAGTTATCGGTAAGTACCACCCGCACGGTGACTCAGCGG
TTTACGAATCAATGGTCAGAATGGCGCAGGATTTTAACTACCGCTACATGCTTGTTGACGGACACGGCAACT
TCGGTTCGGTTGACGGCGACTCAGCGGCCGCGATGCGTTACACAGAAGCGAGAATGTCAAAAATCGCAATG
GAAATTCTGCGTGACATTACGAAAGATACGATTGATTATCAAGATAACTATGACGGCGCAGAAAGAGAACCT
GTCGTCATGCCTTCGAGATTTCCGAATCTGCTCGTAAACGGAGCTGCCGGTATTGCGGTCGGAATGGCGACA
AATATTCCTCCGCATCAGCTTGGGGAAGTCATTGAAGGCGTGCTTGCCGTAAGTGAGAATCCTGAGATTACA
AACCAGGAGCTGATGGAATACATTCCGGGCCCGGATTTTCCGACTGCTGGTCAGATTTTGGGCCGGAGCGG
CATCCGCAAGGCATATGAATCCGGACGGGGATCAATCACAATCCGGGCTAAGGCTGAAATCGAAGAGACAT
CATCAGGAAAAGAAAGAATTATTGTTACGGAACTTCCTTATCAGGTGAACAAAGCGAGATTAATTGAAAAAA
TCGCAGATCTTGTCCGAGACAAAAAAATCGAAGGAATTACCGACCTGCGAGACGAATCCGACCGTAACGGA
ATGAGAATCGTCATTGAGATCCGCCGTGACGCCAATGCTCACGTCATTTTGAATAACCTGTACAAACAAACG
GCCCTGCAGACGTCTTTCGGAATCAACCTGCTGGCGCTCGTTGACGGACAGCCGAAGTGCTGAGCCTGAA
GCAATGCCTGAGCCATACCTTGAA
