# Supplementary material for: Bacillus velezensis FX-6 suppresses the infection of Botrytis cinerea and increases the biomass of tomato plants
Source: PLoS One. 2023 Jun 15;18(6):e0286971. doi: 10.1371/journal.pone.0286971 (PMC10270589; doi:10.1371/journal.pone.0286971)
Supplement: Attachment — Submitted filename: Response to Reviewers.docx [file pone.0286971.s003.docx]

Dear reviewers：

Thank you very much for your comments and professional advice. These opinions help to improve academic rigor of our article. Based on your suggestion and request, we have made corrected modification on the revised manuscript. We hope that our work can be improved again. Furthermore, we would like to show the details as follows:

**Reviewer #1（Comments）:**

The abstract of the manuscript is very poorly written with less clarity. I suggest improving it and make it technically strong. The promising results must be briefly explained in the abstract.

**Response:** the abstract of the manuscript has been revised to make the content clarity. The promising results have been briefly explained in the abstract.

Line 25 *B. cinerea* write its full name when using first time.

**Response:** *B. cinerea* has been changed to *Botrytis cinerea*.

Line 29 - 30 This statement is not clear.

**Response:** this statement has been supplemented to make it clear, and added a reference.

Line 37 (Army of attention) what does it mean?

**Response:** an army of attention has been changed to a huge attention.

Line 40 - 41 Statement is very confusing.

**Response:** the content here has been revised to make the expression clear.

Line 49 - 52 This part is not clearly written.

**Response:** this part has been rewritten to express clearly.

Line 55 - 57 Add complete address of fungal collection Bank.

**Response:** complete address of fungal collection Bank already added.

Line 58 - 60 Have you confirmed the pathogenicity of the collected fungal agents?

**Response:** yes, I have confirmed the pathogenicity of the collected fungal agents.

Line 55 - 64 The sentences are very confusing. Re-write them.

**Response:** the sentences have been carefully rewritten to make them clear.

Line 72 - 73 Which fungal strains were used in this screening test?

**Response:** the fungal strain used in this screening test was *B. cinerea*, and has been supplemented in the article.

Line 75 At what temperature the plates were incubated and for how many days?

**Response:** the plates were cultured at 25 ℃ for about 6 days, and has been supplemented in the article.

Line 83 How bacterial characters were directly observed? At what lens power cell size and morphology was studied?

**Response:** a single colony was taken and spread evenly on a glass slide and then dry-fixed and Gram-stained for 1 min. Next, the morphology, cell size, and spore characteristics of strain FX-6 was observed under a microscope.

Line 101 - 105 Why was this test done for FX-6 when screening test was already done? Furthermore, there is no information on the No. of replications and how inhibition zone was measured?

**Response:** the test here is the antagonistic activity of FX-6 against other seven pathogenic fungi, so as to determine the inhibition spectrum of FX-6. The No. of replications is three. The inhibition zone is not measured, we calculated the inhibitory rate by measuring the colony diameter.

Line 106 - 115 The given information is very confusing. Why fermentation time finding related test was only performed against *B. cinerea* only?

**Response:** this study is aimed at screening biocontrol bacteria for tomato gray mold, so the fermentation time finding related test was only performed against *B. cinerea*.

Line 116 - 122 Was it a detached leave assay? Why only *B. cinerea* was selected for this study? Where were the leaves cultured? If it was a detached leaf assay, then how leaves were not wilted up to 5 days?

**Response:** yes, it was a detached leave assay. Because my study is aimed at screening biocontrol bacteria for tomato gray mold, so only *B. cinerea* was selected for this assay. The petioles of isolated leaves were wrapped with soaked cotton, and then placed in a petri dish with wet filter paper. The cotton balls and filter paper were moist during the whole test process, which ensured the leaves were not wilted up to 5 days.

Line 144 What was the incubation period? It indicates that it has phosphorous solubility …….. Delete it.

**Response:** the expression of the incubation period is incorrect and has been deleted. The sentence “It indicates that it has phosphorescent solubility...” has been deleted.

Line 148 Write down the full name of DF salt minimal medium.

**Response:** the full name of DF salt minimal medium is Dworkin and Foster (DF) salt minimal medium, and has been added.

Line 152 - 160 How same sized tomato seeds were selected? Where were the treated seeds placed before measuring germination percentage etc.? What was the size of the pot used and quantity of the soil? Was it a replicated trial?

**Response:** just look at the same size with eyes. The treated seeds were placed in a petri dish with wet filter. The diameter of pot was 20 cm, the height was 15 cm, and the quantity of soil was about 1.6 kg. It was a replicated trial, and each treatment was repeated three times.

Line 166 - 174 Why only FX – 6 was selected for further studies whereas many other strains were almost equally effective against *B. cinerea*. Mention there antagonistic activity against other fungal isolates as well.

**Response:** compared with other strains, strain FX-6 has the highest antagonistic activity against *B. cinerea*, and the antagonistic activity did not significantly decrease from the first generation to the tenth generation. The antagonistic activity against other fungal isolates was the content of the inhibitory spectrum of strain FX-6 later, so it should not be mentioned here.

Line 183 - 188 The results are not properly explained. Why only FX – 6 was selected to test against other pathogenic fungal agents?

**Response:** because strain FX-6 has the highest antagonistic activity against *B. cinerea*, and the antagonistic activity did not significantly decrease from the first generation to the tenth generation. Therefore, only FX-6 was selected to test against other pathogenic fungal agents.

Line 189 Table 2 Inhibition rates of strain F3A against seven pathogenic fungi ….. It should be cross checked.

**Response:** I have carefully checked and corrected the mistakes found.

Line 199 - 206 The results are not properly explained. Thins are very confusing.

**Response:** the results here have been revised.

Line 218 - 223 The results are not properly explained.

**Response:** the results here have been revised.

Line 233 Table 4. Fresh weight（cm）…… Cross check the Unit. What is CK in the table?

**Response:** the fresh weight unit is wrong and has been corrected. CK in the table is the seeds treated with water.

Discussion portion is very poor. The results are not properly discussed with already published literature.

**Response:** the discussion portion has been revised, and new references have been added.

Line 280 The study does not reflect claim to identify a new bacteria agent as an antagonist. This objective is not mentioned anywhere in the manuscript.

**Response:** this expression is wrong and has been corrected.

Conclusion needs to be more eye catching and logical.

**Response:** the conclusion has been revised.

**Reviewer #2** **(Comments):**

1.The title can be modified as “*Bacillus velezensis* FX-6 suppresses the infection of *Botrytis cinerea* and increases the biomass of tomato plants”

**Response:** the title has been modified as “*Bacillus velezensis* FX-6 suppresses the infection of *Botrytis cinerea* and increases the biomass of tomato plants”.

2. Line 9: Replace “*Botrytis cinerea*-caused tomato gray” with “*Botrytis cinerea* causing tomato gray”

**Response:** “*Botrytis cinerea*-caused tomato gray” has been changed to “*Botrytis cinerea* causing tomato gray”.

3. Line 11: While mentioning the code of the strain for 1st time please write the name of the species

**Response:** the species name *Bacillus velezensis* has been added before FX-6.

4. Line 25: Replace “is one of the countries with the largest area of production worldwide” with “is one of the countries with the largest cultivated area worldwide”

**Response:** “is one of the countries with the largest area of production worldwide” has been changed to “is one of the countries with the largest cultivated area worldwide”.

5. Line 36-37: Replace “*Bacillus* strains have received an army of attention as biocontrol agents” with “*Bacillus* strains have received a huge attention as biocontrol agents”

**Response:** “*Bacillus* strains have received an army of attention as biocontrol agents” has been changed to “*Bacillus* strains have received a huge attention as biocontrol agents”.

6. Line 40-41: Replace “Up to now, there are just several reports on the control of tomato gray mold by *B. velezensis*” with “There are several reports on the control of tomato gray mold by *B. velezensis*”

**Response:** “Up to now, there are just several reports on the control of tomato gray mold by *B. velezensis*” has been changed to “There are few reports on the control of tomato gray mold by *B. velezensis*”.

7. It is better to replace the word biocontrol fungicide with biopesticide

**Response:** the word biocontrol fungicide has been changed to biopesticide.

8. Language needs improvement throughout the manuscript

**Response:** the language of the manuscript has been improved.

9. The result representation is very weak. The tables should be replaced with high resolution graphs

**Response:** the result representation has been revised. The tables have been replaced with high resolution graphs.

10. Discussion must be improved and supported with more references and logical reasoning.

**Response:** The discussion has been revised and new references have been added.

11. Manuscript needs formatting according to journal’s format.

**Response:** Manuscript has been formatting according to journal’s format.

Thank you very much for your attention and time. Look forward to hearing from you.

Yours sincerely,

Zhaoyu Li
